# Supplementary material for: Clinically Translatable Approaches of Inhibiting TGF-β to Target Cancer Stem Cells in TNBC
Source: Biomedicines. 2021 Oct 4;9(10):1386. doi: 10.3390/biomedicines9101386 (PMC8533357; doi:10.3390/biomedicines9101386)
Supplement: Supplementary file 1 [file biomedicines-09-01386-s001.zip › biomedicines-1384457-supplementary.pdf]

Supplemental Table S1: Summary of the Papers Describing the Biphasic Role of TGF-β Signaling

Tumor suppressive roles of TGF-β	
**Study**	**Findings**
Chaudhury, A.; Howe, P.H.J.I.l. [15]	TGF-β acts as a tumor suppressor (via induction of p21CIPI and suppression of c-Myc in HaCaT cells)
Yoo *et al* [22]	TGF-β stimulates apoptosis (via SMAD dependent activation of GADD45b, which activates p38 based programmed cell death)
Huynh et al [23], Spender et al [24], Ozaki et al [25], & Wiener et al [26]	TGF-β modulates apoptosis via regulation of pro-apoptotic (BIM and BIK) and anti-apoptotic (Bcl-2) factors
Tang et al [27] & Sikder et al [28]	TGF-β downregulates ID1 to induce differentiation in xenograft models of early-stage breast cancer
Tang et al [27]	Transfection of dominant negative type II TGF-β receptors resulted in 10-20x more effective tumor formation compared to MCF10A-Ca1h xenograft controls (xenograft model of early stage breast cancer)
Ji et al [36]	TGF-β addition induced expression of p21WAF1 and suppressed growth and migration in control cells compared to non-small-cell lung carcinoma cells and mouse oral cancer-derived cells transfected with mutant p53

Oncogenic roles of TGF-β	
**Study**	**Findings**
Ó hAinmhire et al [33]	TGF-β induced migration and proliferation in ovarian cancer cell line models with loss or mutation of p53
Ji et al [36]	Transfection of mutant p53 binds MH2 domain in SMAD3-4 complex, explaining the reduced responsiveness to TGF-β and increased migration and proliferation in non-small-cell lung carcinoma and mouse oral cancer-derived cells<br><br>TGF-β stimulation decreased expression of p21 and p15 tumor suppressors in non-small-cell lung carcinoma cell line with mutant p53 while increasing the expression of MMPs and Slug for increased cellular migration
Conery et al [34]	Dysregulated P13K/Akt signaling translocated SMAD3 outside the nucleus and halted TGF-β mediated apoptosis
David et al [35]	Oncogenic role:<br>TGF-β through SMAD4 stimulates EMT and migration<br><br>Tumor suppressive role:<br>TGF-β upregulates SNAI1, which inhibits KLF5 allowing for SOX4 levels to increase and induce apoptosis

Supplemental Table S2: Summary of the papers referenced in Section 1.4: Clinical Importance of CSCs in TNBC

**Study**	**Mechanism of CSCs in TNBC**	**Tumor type**
Al Hajj *et al* [55]	$CD44^+/CD24^-$ CSC population demonstrated a 100 fold increased tumorigenicity via serial dilution assays in mammary fat pad transplantation compared to unfractionated tumor cells.	Breast cancer
Creighton *et al* [66]	Post chemotrophic breast cancer cells express increased $CD44^+/CD24^-$ CSCs compared to pre-treatment. Increased expression of FN1, SNAI2, VIM, FOXC2, MMP2, and MMP3 (mesenchymal related genes) as well as diminished CDH1 (an epithelial related gene) suggest enhanced mesenchymal and EMT properties.	Breast cancer
Yu *et al* [71]	Pre- and post- chemotherapy analysis in breast cancer CSCs resulted in finding that chemotherapy-responsive patients were correlated with decreased CSCs and decreased in mesenchymal to epithelial CSC populations. On the other hand, progressive disease was associated with increased mesenchymal CSCs and increased multicellular CSC clusters with high mesenchymal markers promoting tumor cell dissemination.	Breast cancer
Papadaki *et al* [72]	Breast cancer cells with hybrid epithelial/mesenchymal CSCs were associated with increased lung metastasis, relapse and decreased progression-free survival. Post-chemotherapy cells expressed increased numbers of hybrid epithelial/mesenchymal CSCs while epithelial and mesenchymal CSCs were reduced. This suggests that chemotherapy alters plasticity and population dynamics, decreasing	Breast cancer

	patient prognosis and rates of relapse by altering CSC population.	
Sulaiman *et al* [70]	post-chemotherapy exposure led to increased $CD44^{+}/CD24^{-}$ and $ALDH^{high}$ CSC populations which correlated with increased tumorigenicity *in vivo* post therapy (forming tumors at a rate of 80% upon injection of 1,000,000 cells versus the control which formed tumors at a rate of 20% with an injection of 1,000,000 cells)	Breast Cancer

Supplemental Table S3: Summary of the Preclinical TGF-β Inhibitors referenced in Section 1.5: TGF-β as a Therapeutic Target to Inhibit TNBC and its CSC population

Study	Inhibitor	Mechanism of Action	Tumor Type
Bhola *et al* [74]	LY2157299	Is a TGF-β type I receptor kinase inhibitor that suppressed tumorigenesis, and inhibited epithelial /mesenchymal CSC populations in paclitaxel treated cell TNBC.	TNBC
Zhu *et al* [78]	Ophiopogonin D	An anti-inflammatory agent that disrupts TGF-β1 stimulation of ITGB1/ FAK/Src/AKT/β-catenin signaling pathway. Thus suppressing TGF- β1 mediated invasion, resistance and metastasis.	TNBC
Sun *et al* [79]	Tunicamycin	A N-linked glycosylation inhibitor that potentially inhibits the TGFβR2 glycosylation necessary for signal sensitivity, interaction with TGFβR1, SMAD2 phosphorylation and ultimately TGFβ signaling in radiotherapy treated ALG3-overexpressed breast cancer.	Breast cancer
Sun *et al* [79]	LY2109761	A TGFβR2 inhibitor that suppressed cancer stemness, induced post-radiotherapy apoptosis and formation of CD44+/CD24- CSCs in radiotherapy treated ALG3-overexpressed breast cancer.	Breast cancer

Schech *et al* [82]	Entinostat	A class I HDAC inhibitor with TGF-β modulating properties inhibits $CD44^+/CD24^-$ CSCs in TNBC and formation of mammosphere in immortalized non-cancerous breast cancer cell lines.	TNBC
Wahdan-Alaswad *et al* [84]	Metformin w/ LY2197299	Metformin (an AMPK activator used to treat type II diabetes) when combined with LY2197299 (a selective TGF-β Receptor I-Kinase Inhibitor) worked synergistically in inhibiting phospho-Smad2 and phosphor-Smad3 protein expression involved in TGF-β1 signaling. Thus suppressed TGF-β1 induced motility and cell invasion in TNBC models.	TNBC
